# Reduction in radioactive internal contamination by ^99m^Tc among medical personnel in nuclear medicine facilities with the use of respiratory tract protection measures

**DOI:** 10.1007/s10967-022-08639-w

**Published:** 2022-11-14

**Authors:** Kamil Brudecki, Eliza Borkowska, Krzysztof Gorzkiewicz, Magdalena Kostkiewicz, Ryszard Misiak, Tomasz Mróz, Ewa Nalichowska

**Affiliations:** 1 Institute of Nuclear Physics, Polish Academy of Sciences, Radzikowskiego 152, 31-342 Kraków, Poland; 2 Nuclear Medicine Department, John Paul II Hospital, Prądnicka 80, Kraków, Poland; 3 Heart and Vascular Diseases Department, Faculty of Medicine, Institute of Cardiology, Jagiellonian University, Collegium Medicum, Prądnicka 80, 31-202 Kraków, Poland; 4 Institute of Physics, Jagiellonian University, Łojasiewicza 11, 30-348 Kraków, Poland

**Keywords:** ^99m^Tc, Internal contamination, Medical personnel, Blood, Lung, Perfusion, Ventilation, Scintigraphy

## Abstract

The main objective of the present publication was to assess the reduction of internal radioactive contamination with ^99m^Tc among medical personnel of nuclear medicine facilities using generally available respiratory tract protection systems. During the current research project, four respiratory tract protection systems were tested by estimation of ^99m^Tc activity levels in blood samples collected from medical personnel. Medical staff were equipped with a disposable surgical mask, a half mask with gas absorbers, a half mask with aerosol absorbers and a half mask with gas absorbers with added Petryanov filter. The presented results indicate that wearing only a disposable surgical mask may significantly reduce radioactive internal contamination among medical personnel and improve their safety in the workplace. The best results of reduced ^99m^Tc concentration in the blood were achieved by the use of a half mask with gas absorbers with added Pertryanov filters and a half mask with aerosol absorbers, where the reduction factors were estimated at 90% and 80%, respectively. Respiratory tract protection systems should become standard equipment for medical personnel performing ventilation–perfusion SPECT lung scans.

## Introduction

^99m^Tc is widely used in single-photon emission computed tomography (SPECT) imaging. One of the medical tests using ^99m^Tc is ventilation–perfusion SPECT lung scans. In the perfusion part, the lung blood supply is assessed by intravenous injection of natural blood proteins (albumin) labelled with 150 MBq of ^99m^Tc. To examine the patency of the bronchial tree and lungs, the ventilation part is performed, where gas containing 400 MBq of ^99m^Tc is inhaled by the patient [1]. DTPA (pentetic acid) or Technegas (ultrafine dispersion of ^99m^Tc-labelled carbon particles with a diameter of several dozen nm) are most often used in this procedure [2–4]. During inhalation of the ^99m^Tc-labelled substances by the patient, some activity is released into the room air and becomes a source of radiation burden and internal contamination for the medical personnel performing the tests.

In 2019–2020 ^99m^Tc internal radioactive contamination was assessed among medical personnel who performed ventilation–perfusion SPECT lung scans in the Department of Nuclear Medicine of the John Paul II Hospital in Krakow. Contamination assessments were performed using ^99m^Tc activity measurements in both workplaces (personal aspirators, mobile aerosol sampler) and blood samples collected from medical staff [5, 6]. The conducted assessments revealed that ^99m^Tc activity concentration in the air in the examination room may reach 10 kBq m^−3^ during ventilation–perfusion SPECT lung scans. Considering the duration and character of medical staff work, daily intake caused by ^99m^Tc activity in the air can reach a level of 40 kBq. The obtained results of ^99m^Tc activity in workplaces were convergent with the results of ^99m^Tc activity in blood samples (maximum value 1300 Bq L^−1^ [6]). The similarity of the equipment and procedures used by these medical personnel to those used all over the world allows us to assume that the issue of ^99m^Tc internal contamination during ventilation–perfusion SPECT lung scans is worldwide, which is confirmed by the literature data [7–12].

In an overwhelming number of current nuclear medicine facilities, the basic radiation protection measures for medical personnel working with radiopharmaceuticals are lead aprons and disposable gloves. There are no measures to protect against internal contamination resulting from inhalation of contaminated air. The main objective of the present publication was to assess the potential reduction of internal radioactive contamination with ^99m^Tc among medical personnel of nuclear medicine facilities using generally available respiratory tract protection systems. During the current research project, four respiratory tract protection systems were tested using ^99m^Tc activity measurements in blood samples collected from medical personnel.

## Material and method

### Medical personnel

The present research was performed among the medical staff in the Nuclear Medicine Department, John Paul II Hospital, Krakow, Poland. The department specialises in SPECT diagnostics and is supplied with a Siemens Symbia T16 SPECT/CT hybrid device and Technegas generator manufactured by Cyklomedica. The facility employs three technicians, four nurses and two physicians and annually performs around 400 ventilation–perfusion SPECT lung scans. Usually, during a single working day, four scans are performed. Each lung ventilation and perfusion scintigraphy procedure is performed by a technician and a nurse.

^99m^Tc was prepared individually for each patient just before starting the procedure.

One technician took part in the present research: a woman aged 30 years, weighing.

72 kg and with a height of 160 cm. Her health could be described as very good. The research study was approved by the Bioethics Committee at the Regional Medical Chamber in Krakow, Poland (decision number 92/KBL/OIL/2020, dated 05.06.2020).

### Respiratory tract protection systems

**Fig. 1:**
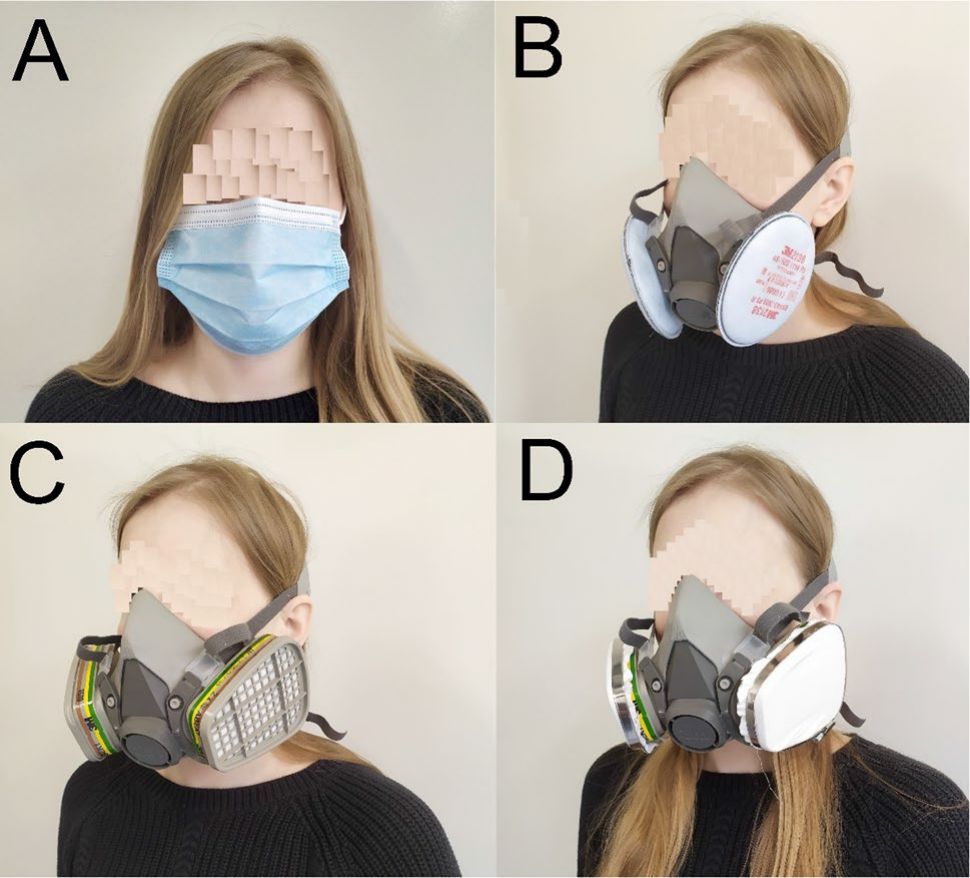
Respiratory tract protection systems used in study (**A**-disposable surgical mask, **B-**half mask with aerosol absorbers, **C**-half mask with gas absorbers, **D**-half mask with gas absorbers with added Petryanov filter FPP-15–1.5)

^99m^Tc activity concentration in blood samples was examined on five working days (at weekly intervals). Each day, medical staff were equipped with a different respiratory tract protection system: a disposable surgical mask, a half mask with gas absorbers, a half mask with aerosol absorbers and a half mask with gas absorbers with added Petryanov filter FPP-15–1.5. A 3 M™ Reusable Twin-Filter Half Mask 6000 Series was used with different mounted filters. Depending on the mounted filters, it can protect against hazardous vapours, gases and particulates and is commonly used in the construction, chemical and pharmaceutical industries. A 3 M™ filter 2138 protects against solid and liquid particles, ozone and nuisance-level gases and vapours. A 3 M™ filter 6059 protects against a range of gases and vapours. The additional Petryanov filter FPP-15–1.5 (polyvinyl chloride) was manufactured by ESFIL TEHNO AS (Estonia). These filters are commonly used for aerosol collection because of their high collection efficiency (up to 97%) [13]. Figure 1 presents the equipment used. During the last day, personnel were working without any respiratory protection system (which is currently standard) to estimate reference levels. During each day of the research campaign, medical personnel performed the same number of ventilation–perfusion SPECT lung scans, i.e. four.

The medical crew was trained in the use of the half-masks by the hospital health and safety inspector in accordance with the guidelines described in the user manual and instructional videos provided by the masks’ manufacturer.

### Blood sampling and measurement

Blood samples were drawn over five days, when ventilation–perfusion lung scans were performed. Every day of the experiment, six blood samples of 10 mL each were collected. The blood samples were drawn every ca. 1 h while ventilation–perfusion SPECT lung scans were performed. Overall, ^99m^Tc activity was determined in 30 blood samples.

The activity concentration measurements were performed using a gamma-ray spectrometer equipped with an HPGe detector (P-type) with 10% relative efficiency.

A germanium detector was surrounded by a 5-cm standard lead shield. Data acquisition was performed using an ORTEC 919E Ether NIM multichannel analyser, and spectral analysis used ORTEC Maestro software. The spectrometry system was designed and developed at the Institute of Nuclear Physics PAN.

The drawn blood samples were transferred into cylindrical disposable measuring vessels 50 mm in diameter and 5 mm high. The disposable measuring vessels had the same geometry as calibration sources for the efficiency calibration of the spectrometer (multi gamma source SZN 40/10 by Polatom). The activity of ^99m^Tc was calculated based on analysis of 140.5 keV gamma-line (intensity 88.5%) [14]. The measurement time depended on the activity of the sample: the most active samples were measured for only 10 min. The obtained results were corrected on sampling time. The uncertainty of the presented results is at the level.

of 1 sigma.

## Results and discussion

**Fig. 2:**
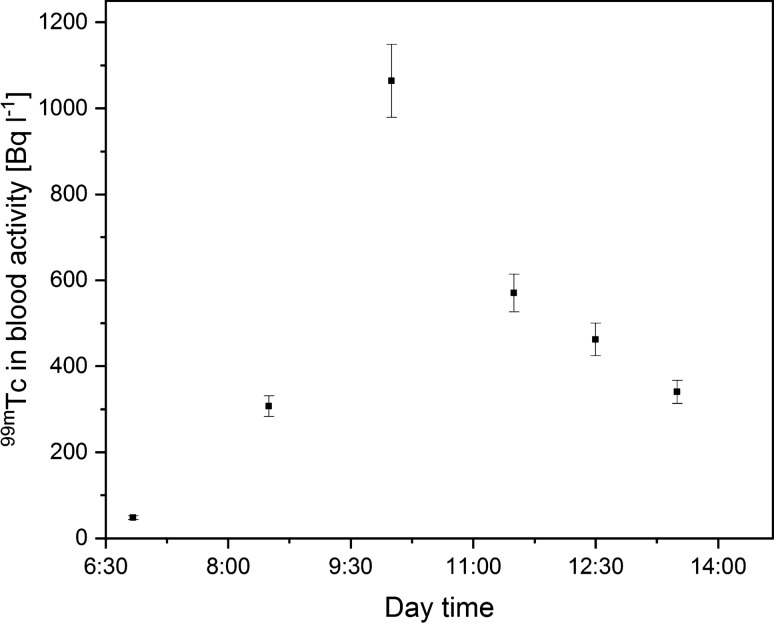
Technician ^99m^Tc blood concentration during the workday without any respiratory tract protection measure (one sigma measurement error)

^99m^Tc activity concentration in the blood samples was examined during five working days (at weekly intervals). Each day, medical staff were equipped with another respiratory tract protection system. In the first case, the technician who conducted the scans did not wear any respiratory tract protection measures. The highest ^99m^Tc concentration in the blood reached 1,064 ± 65 Bq L^−1^. Figure 2 shows the changes in ^99m^Tc concentration during the workday, which were in accordance with previously conducted research [6]. These values are treated as a reference level of radioactive internal contamination.

**Fig. 3:**
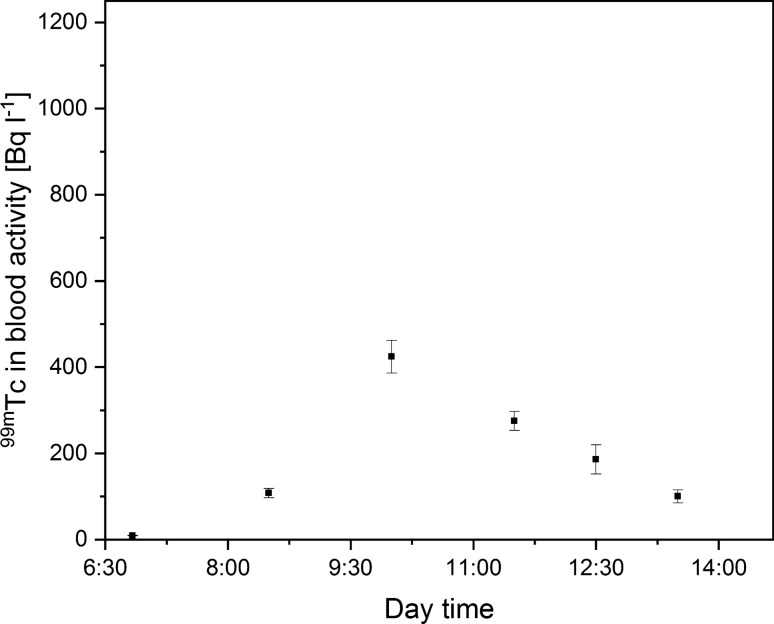
Technician ^99m^Tc blood concentration during the workday with the use of disposable surgical mask (one sigma measurement error)

Next, the usage of disposable surgical masks significantly reduced the maximal concentration of ^99m^Tc in the blood to the level of 424 ± 38 Bq L^−1^ (Fig. 3). The reduction factor reached around 60% compared to the reference level. Notably, during the COVID-19 pandemic, medical staff working in hospitals in Poland were obligated to wear surgical masks during the entire working day. The presented results indicate that an additional healthcare advantage was the reduction of radioactive internal contamination among radiation facility medical crew.

**Fig. 4:**
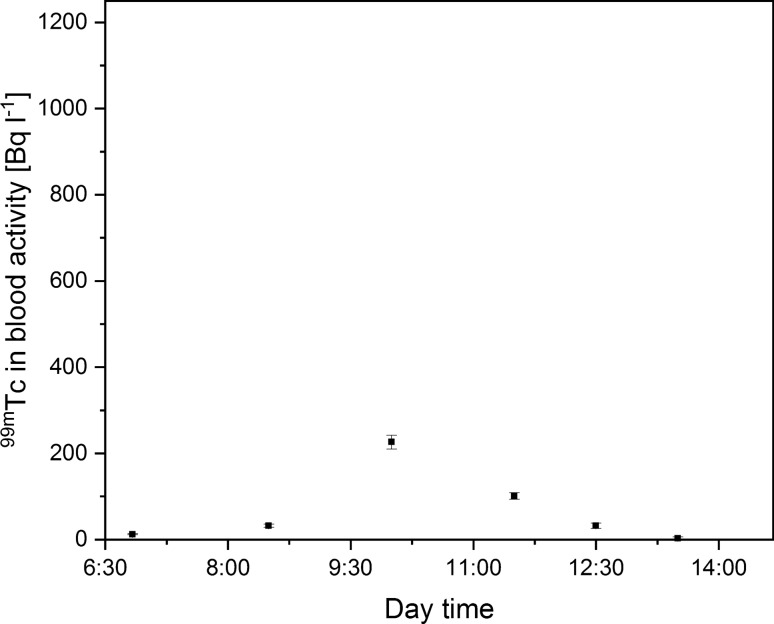
Technician ^99m^Tc blood concentration during the workday with the use of half mask with aerosol absorber (one sigma measurement error)

**Fig. 5:**
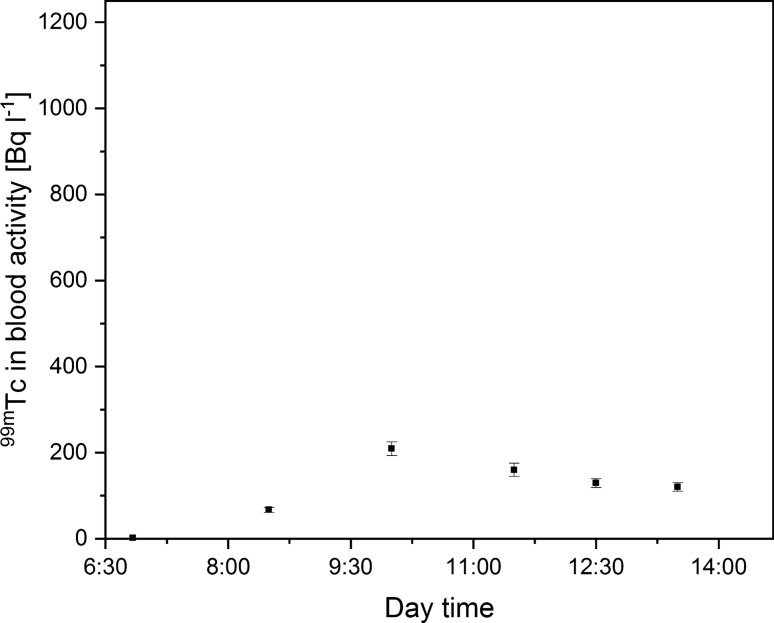
Technician ^99m^Tc blood concentration during the workday with the use of half mask with gas absorber (one sigma measurement error)

The next tested respiratory tract protection measure was a half mask with aerosol and gas absorbers. In both cases, the ^99m^Tc concentrations were similar,

namely 226 ± 16 Bq L^−1^ and 209 ± 16 Bq L^−1^ for the half mask with aerosol and gas absorbers, respectively. However, the change in ^99m^Tc blood concentration over the day for these protection systems was different. The decrease of ^99m^Tc concentration over time was much slower in the case of the half mask with gas filter (Fig. 4, Fig. 5). The explanation for this phenomenon is that Technegas, used during ventilation–perfusion SPECT lung scans, consists of carbon aerosols that do not attach permanently to active charcoal particulates in filters, leading to its transmission to the respiratory tract and consequently increased concentration of ^99m^Tc in the blood. Considering the maximal activity concentration of ^99m^Tc in blood samples collected when the medical staff member was wearing a half mask, the internal radioactive contamination reduction factor reached the level of 80%.

**Fig. 6:**
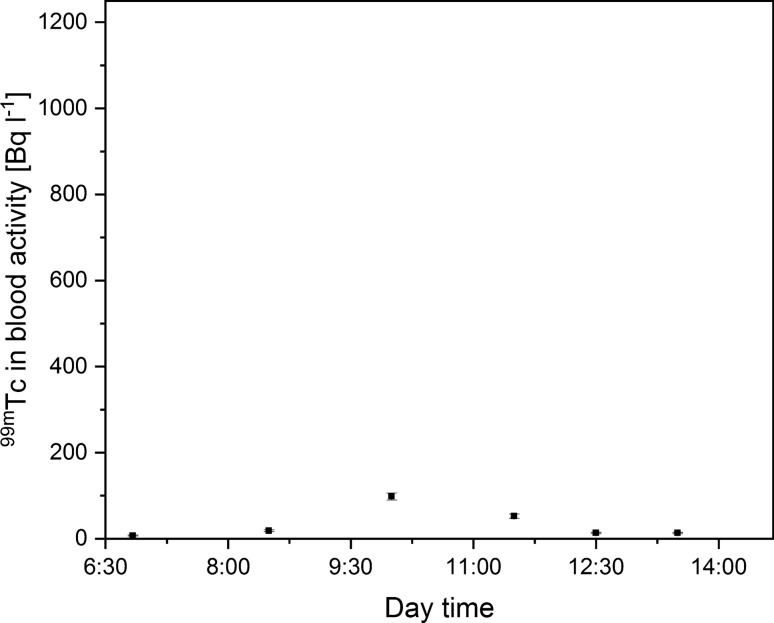
Technician ^99m^Tc blood concentration during the workday with the use of gas absorber with added Petryanov filter (one sigma measurement error)

Furthermore, the best results were obtained by the application of a half mask with gas absorbers with added Petryanov filters. The multi-layer construction of the filter media provided a ^99m^Tc concentration reduction of up to 91% compared to the reference level. The maximal activity reached only 98 ± 8 Bq L^−1^. Figure 6 depicts the change of ^99m^Tc blood concentration during the workday.

The graphs show that, regardless of the type of mask, the maximum concentration of ^99m^Tc appeared at a similar time (around 10:00). The concentration reached its maximum shortly after the second treatment of the day. The metabolic processes seem to have the greatest influence on it. ^99m^Tc is cleared from the blood very quickly. The biological half-life in blood is only around 10 min [15]. The relatively short ^99m^Tc physical half-life also helps in this process.

## Conclusions

In an overwhelming number of current nuclear medicine facilities, the basic radiation protection measures for medical personnel working with radiopharmaceuticals are lead aprons and disposable gloves. No measures protect against internal contamination resulting from the inhalation of radioactively contaminated air. The presented results indicate that wearing only a disposable surgical mask may significantly reduce radioactive internal contamination among medical personnel and improve their safety in the workplace. The best results of ^99m^Tc concentration reduction in the blood were achieved by the use of a half mask with a gas absorber and Pertryanov filters. However, this method needs further development and improvement because the Pertryanov filter medium is not intended for human respiratory tract protection. The greatest danger comes from loosely attached PCV fibres that may enter the respiratory tract during inhalation and cause undesirable effects.

The second-best method, a half mask with aerosol absorbers, gave comparable results in the reduction of internal contamination (80%) without introducing possible hazards. Importantly, the presented measures of respiratory tract protection are economical, generally available and easy to handle.

To sum up, at least one of the tested protection systems should become standard equipment for medical personnel performing ventilation–perfusion SPECT lung scans.
